# A Practical Treatise on the Management and Diseases of Children

**Published:** 1843-07

**Authors:** 


Art. IV.—*A Practical Treatise on the Management and Diseases of Children.* By Richard T. Evanson, M.D., and Henry Maunsell, M.D. Second American, from the fourth Dublin edition, with notes, by D. F. Condie, M.D. Philadelphia: Barrington and Haswell, 1843.

The extraordinary success of this work is the best proof of its intrinsic merits. The patronage which it has obtained in Great Britain is greater than was ever conferred upon any other book on the Diseases of Children; a large edition was rapidly disposed of in this country; and its translation into the German language shows how it is appreciated in the north of Europe. The present edition, from the fourth Dublin, has been enriched with notes by Dr. Condie, a learned physician of Philadelphia, who is practically conversant with whatever pertains to the subject. The most valuable additions by the editor relate to gangrene of the mouth, and cholera infantum, two topics which were very imperfectly discussed by the authors, and a knowledge of which is of paramount importance to the American practitioner. This is especially true of the latter malady, which destroys annually an immense number of children throughout the United States. Gangrene of the mouth is comparatively rare, but it is always dangerous, and generally extremely difficult to manage. It is on this account that we esteem the labors of the American editor as peculiarly valuable, and we only regret that his additions are not more extensive. As it now presents itself, the work of Dr. Evanson and Dr. Maunsell constitutes the best practical treatise on the subject in any language. It embraces a complete outline of the existing state of the science, written in a plain and perspicuous style, and is perhaps as faultless as it is possible for any production of the kind to be. The work is well got up, and reflects much credit upon the enterprising publishers.

